# The effects of different types of Tai Chi exercises on motor function in patients with Parkinson's disease: A network meta-analysis

**DOI:** 10.3389/fnagi.2022.936027

**Published:** 2022-08-29

**Authors:** Honghui Lei, Zhen Ma, Kexin Tian, Ke Liu, Jiaying Wang, Xiangyu Zhu, Baohong Mi, Ying Chen, Qihao Yang, Huili Jiang

**Affiliations:** ^1^Department of Rehabilitation, School of Acupuncture-Moxibustion and Tuina, Beijing University of Chinese Medicine, Beijing, China; ^2^Department of Encephalopathy, Beijing Dongzhimen Hospital, Beijing University of Chinese Medicine, Beijing, China; ^3^Department of Sport Rehabilitation, School of Exercise and Health, Shanghai University of Sport, Shanghai, China

**Keywords:** Tai Chi, Parkinson's disease, exercise therapy, movement disorders, network meta-analysis

## Abstract

**Background:**

Tai Chi can show improvement in balance and motor ability of elderly patients with PD. However, there were few reports on differences in outcomes associated with different types of Tai Chi on improving exercise capacity in elderly patients with PD. We compared the improvement of motor function in Parkinson's patients with different types of Tai Chi, for finding an optimal intervention.

**Methods:**

The following databases were searched from the beginning of the establishment of each database to 10 January 2022: PubMed, EMBASE, The Cochrane Library, CNKI, Wanfang Database, and VIP Database. Randomized controlled trials incorporating different types of Tai Chi for PD were included. The outcome measures were UPDRSIII and BBS. NMA was conducted using Stata 15.0 based on a frequentist framework.

**Results:**

A total of twenty trials were eligible, including 996 participants. In conventional meta-analysis, as for the UPDRSIII scale, 24-form simplified Tai Chi (SMD = −1.272, 95% CI [−2.036, −0.508], *P* < 0.05, I^2^ > 50%), Tai Chi exercise program (SMD = −0.839, 95% CI [−1.828, 0.151], *P* > 0.05, I^2^ > 50%), 8-form simplified Yang style Tai Chi (SMD = −0.325, 95% CI [−1.362, 0.713], *P* > 0.05, I^2^ > 50%), and 8-form simplified Chen style Tai Chi (SMD = −0.28, 95% CI [−0.97, 0.42], *P* > 0.05, I^2^ > 50%) were statistically more efficient than the control group. For BBS outcome, 24-form simplified Tai Chi (MD = 3.979, 95% CI [3.364, 4.595], *P* < 0.05, I^2^ <50%), Tai Chi exercise program (MD = 5.00, 95% CI [2.07, 7.93], *P* > 0.05, I^2^ > 50%), and 8-form simplified Chen style Tai Chi (MD = 1.25, 95% CI [0.52, 1.98], *P* < 0.05, I^2^ > 50%) were better than the control group. In the network meta-analysis, the results of UPDRSIII were as follows: 24-form > TCEP > 8-form YS > 8-form CS > control. The ranking probability of BBS was as follows: TCEP > 24-form > 8-form CS > control.

**Conclusion:**

Among the four treatments studied, 24-form Tai Chi and Tai Chi exercise programs have shown better efficacy than other types. Our study provides new insights into exercise therapy for PD and may contribute to the formulation of a clinical exercise prescription.

**Systematic review registration:**

Identifier: CRD42021285005.

## Introduction

Parkinson's disease (PD) is a common chronic, debilitating neurodegenerative disease, affecting more than 2% of the population aged above 65 years. The mechanism of pathogenesis stems from the striatal deficiency of the neurotransmitter dopamine due to the selective loss of dopaminergic neurons in the substantia nigra pars compacta of the midbrain (Cuenca et al., 2019; Hatano et al., 2022). The neurodegenerative disorder could seriously affect the physical, psychological, and social health of patients (Ascherio and Schwarzschild, 2016). The cardinal features of PD are motor symptoms such as tremors, rigidity, akinesia, and postural instability (Samii et al., 2004; Balestrino and Schapira, 2020). As the condition advances, PD would impose a great burden on a patient's life and recovery.

The management of PD in most treatment guidelines recommends 2 therapeutic approaches, including pharmaceutical therapy and nonpharmaceutical therapy (Alonso-Frech et al., 2011). Exercise therapy is an effective nonpharmacological therapy for patients with earlier and moderate disease (Yu et al., 2018). It could induce brain plasticity, increase synaptic strength, and potentiate functional circuitry (Petzinger et al., 2013). A study showed that to reduce the loss of dopaminergic neurons and play a neuroprotective role, exercise could allow the surviving dopaminergic neurons an increase in the dopamine response or promote their synthesis of dopamine (Su, 2020). In addition, exercise might have a beneficial effect on the activation of neurogenesis (Fabel et al., 2003; Yasuhara et al., 2007). There is growing evidence that several classes of brain-derived growth factors increased after exercise might mediate the effects of exercise on learning, neurogenesis, and angiogenesis (Trejo et al., 2001). In a systematic review (Ni et al., 2014), Tai Chi, a traditional Chinese exercise, combined with drug therapy can result in significant efficacy in improving motor symptoms, balance, mobility, and stride length in individuals with PD. A study (Vergara-Diaz et al., 2018) evaluated the impact of TC while participants were in a PD off-medication state and found a benefit of TC in reducing dual tasking stride time variability. A meta-analysis (Song et al., 2017) supported the efficacy of TC for reducing falls and improving balance for patients with PD.

To date, there have been several types of Tai Chi that are usually used in the treatment of PD. According to current clinical studies, we found that 24-form simplified Tai Chi is the most commonly used, followed by Tai Chi exercise program, 8-form simplified Yang style Tai Chi, and 8-form simplified Chen style Tai Chi. However, no adequate evidence has indicated which type of Tai Chi was recognized as the most effective form suitable for patients with PD to improve motor function. Therefore, our study conducted a network meta-analysis (NMA) to explore which type of Tai Chi has the best impact on the effectiveness in improving movement disorders for treating PD, with stretching or walking as the reference group.

## Methods

### Protocol and registration

This systematic review and network meta-analysis were strictly conducted according to the PRISMA-NMA (The Preferred Reporting Items for Systematic Review and Meta-analysis for Network Meta-analysis) statement (Hutton et al., 2015). Moreover, we have registered the protocol of this review in PROSPERO (CRD42021285005) in advance.

### Eligibility criteria

#### Inclusion criteria

The inclusion criteria were as follows: full text obtainable in Chinese or English; elderly patients who meet the internationally recognized diagnostic criteria for PD, regardless of age, sex, courses of disease, or sources of cases; the study design restricted to randomized controlled trials (RCTs); and comparisons between interventions of 24-form simplified Tai Chi (24-form), Tai Chi exercise program (TCEP), 8-form simplified Yang style Tai Chi (8-form YS), 8-form simplified Chen style Tai Chi (8-form CS), and a control group.

Regarding the 24-form, it was mainly adapted by China National Sports Commission based on the traditional Yang style Tai Chi (China Sports Editorial Board., 1980). The actions lessened the fighting skills of traditional Tai Chi and emphasized the standardization of each movement so that the 24-form was more suitable for promotion. It is the most popular in the world now.

Regarding the 8-form YS, it was developed based on the traditional Yang style Tai Chi, and the core program consisted of five forms of Tai Chi-based stepping exercises, which were as follows: moving hands like clouds, parting the wild horse's mane, stepping up and thrusting downward, repulsing monkeys, and grasping the peacock's tail. A total of two trials were included in this study. One used the movement design of Li et al. (2003), and the other used the first 8 formulas of 24-form simplified Tai Chi.

Regarding the 8-form CS, its movements developed based on the traditional Chen Tai Chi style, including commencing form, parting the wild horse's mane, six sealing and four closing, moving hands like clouds, pat a high horse, step back and ride, warrior pounds the mortar, and closing form. This study included an 8-form simplified Chen-style Tai Chi trial.

One significant difference between the 8-form CS and 8-form YS is that the Chen style is characterized by controlled actions/motions, while the Yang style movements are evenly paced. In addition, there are differences in the postures or forms included, the order of their appearance, and the level of difficulty of movements (Li et al., 2003).

Finally, it is regarding TCEP. The trials would be classified as TCEP when patients with PD participated in Tai Chi exercises specially formulated by researchers to treat PD motor symptoms. For example, 2 studies drew on the classic movements in Tai Chi routines and then improved and innovated the exercise program in a targeted manner (Luan, 2020; You and She, 2020). In the program, 4 studies only selected individual movements of Tai Chi routines, and the patients would practice Tai Chi in combination with warm-up and relaxation exercises (Choi et al., 2013; Chen et al., 2016; Zhu, 2017; Vergara-Diaz et al., 2018).

If participants received the same intervention as the experimental group in addition to Tai Chi, or with the addition of stretching or walking, they were considered the control group.

#### Exclusion criteria

The exclusion criteria were as follows: studies that duplicate published, nonrandomized controlled trials or protocols; using other therapies, such as resistance training or strength training; and studies for which analyzable data were not available, such as those missing the mean and standard deviation (SD).

### Data sources and search strategy

The academic articles were systematically retrieved following the PRISMA-NMA statement. Two reviewers (KX Tian and HH Lei) independently searched 6 databases: PubMed, EMBASE, Cochrane Library, Chinese National Knowledge Infrastructure (CNKI), Wanfang Database, and Chinese Scientific Journal Database (VIP), from their inception to 14 January 2022. Three portions were included in the search terms as follows: (i) Parkinson's disease, Parkinsonian disorders, Parkinsonian syndromes, Parkinsonism, autosomal-dominant Parkinsonism, idiopathic Parkinson's disease, and Lewy body Parkinson's disease; (ii) Tai Ji, Tai Chi, Tai Ji Quan, mind-body therapies, mind-body medicine, exercise movement techniques, pilates-based exercises, martial arts, and Kung Fu; and (iii) randomized controlled trial and randomized placebo. All of the search strategies are documented in the attachment (Supplementary Data sheet 1). To avoid missing eligible trials, ninety-nine cited in the retrieved studies were also retrieved manually.

### Data extraction and quality assessment

Data extraction was performed by two reviewers (HH Lei and Z Ma) who independently extracted data from the remaining RCTs according to predetermined criteria. The name of the first author, year of publication, sample size, gender, age, course of the disease, type of TC treatment, details of control intervention, frequency of TC treatment, duration of TC treatment, and main outcome were recorded for each RCT. If there is incomplete or unclear information in the RCT, the first author should be contacted to obtain relevant data whenever possible. Two reviewers (JY Wang and HH Lei) assessed the quality of included RCTs using the Cochrane risk of bias tool, which focuses on random sequence generation (selection bias), allocation concealment (selection bias), performance bias, detection bias, attrition bias, reporting bias, and other bias. Each bias was judged as low risk, unclear, or high risk in terms of the guidelines. Disagreements were resolved through discussions between the two authors. If the issue still persisted, a third reviewer (XY Zhu) was consulted and then made the final decision.

### Outcome measures

The main results included the MDS Unified-Parkinson Disease Rating Scale (MDS-UPDRS) Part III Motor Function Examination Subscale (MDS-UPDRSIII) (Movement Disorder Society Task Force on Rating Scales for Parkinson's Disease., 2003) and the Berg Balance Scale (BBS) (Berg et al., 1992). The UPDRSIII is one of the most widely used scales for examining the motor function of Parkinson's disease. The motor ability score has 14 items and 28 aspects, each of which is scored from 0 to 4. The lower the score, the better the motor ability. The BBS is one of the most widely used indexes to evaluate balance functions. There are 14 evaluation items, each of which is scored from 0 to 4, with a total score of 56 points. The higher the score, the better the balance function.

### Data synthesis and analysis

Data analysis was performed using Stata 15.0 (Stata Corp, College Station, TX). Considering the wide variation in the UPDRSIII scores across articles, we used the standardized mean difference (SMD) to pool the results. The results of BBS used the mean difference (MD). As for NMA, we calculated SMDs and means with 95% confidence intervals (CIs). The network graphs were constructed to explore comparative relationships among different treatments. First, the pairwise meta-analysis was performed. Then, NMA within the frequentist framework was generated using Stata 15.0. Since the obtained network diagrams were short of closed loops, the consistency model was used for analysis (Yang et al., 2017). Then, we made the NMA interval forest plot. Clinical significance was considered insignificant, if the invalid vertical line intersected the horizontal line of the 95% CI (Li et al., 2017). The surface under the resurfacing curve (SUCRA) was used to reflect the effectiveness rank of the five different treatments. The larger the area under the curve (0–100%), the better the treatment effect. Finally, to investigate the publication bias, this study included the corresponding funnel plots, performing the Egger's test at a significance level of 0.1. The stability of the results is reliable when the funnel plots for most of the included studies are vertically distributed to the midline (x = 0) (Egger et al., 1997).

## Results

### Study selection and characteristics

**Figure 1:**
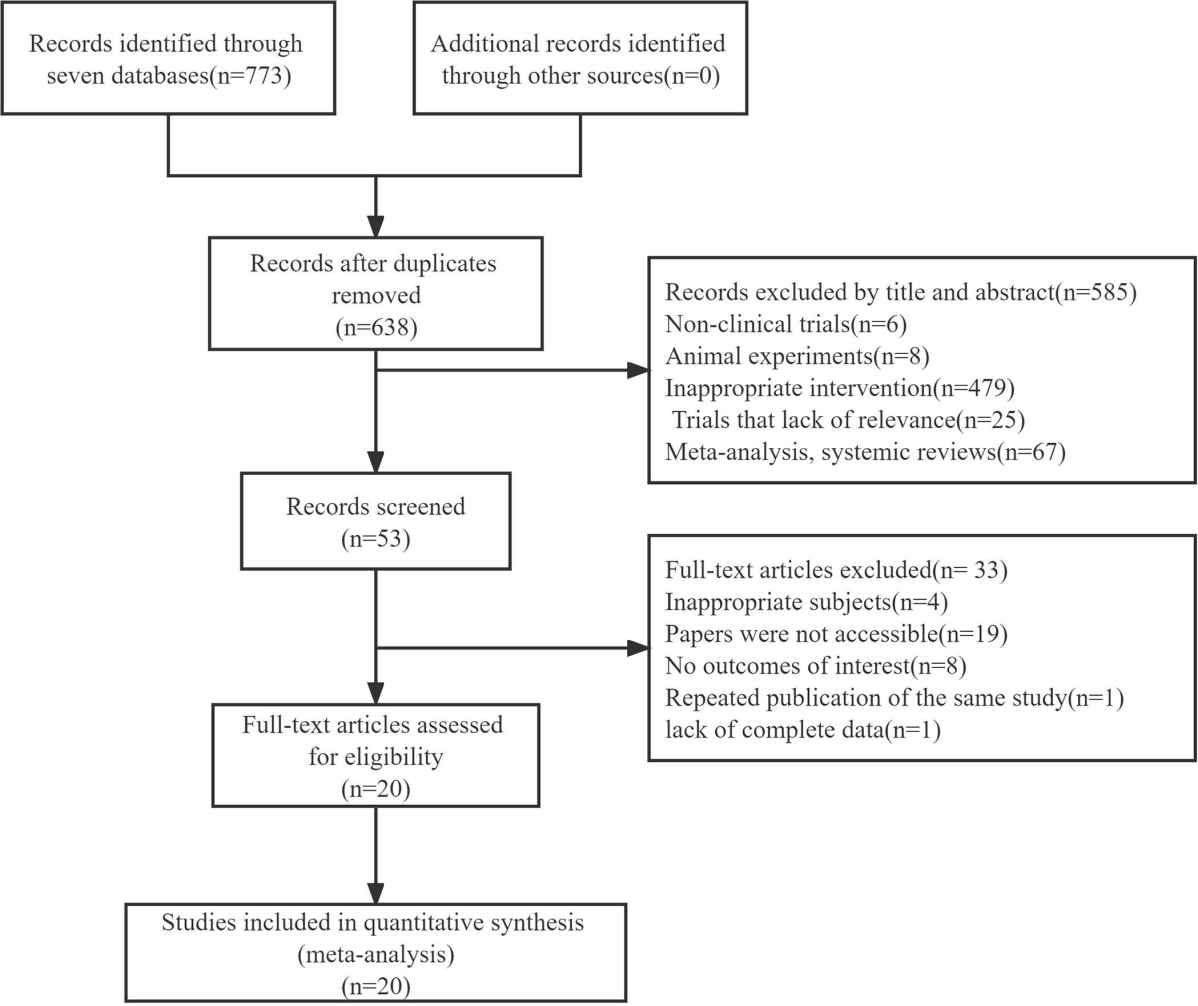
Schematic illustration of the literature search and study selection criteria.

A total of 773 studies were retrieved from the seven electronic databases. After removing duplicates, 638 articles were included and 585 pieces of literature were excluded for not qualifying the inclusion criteria following a review of the titles and abstracts. A full review of the remaining 53 articles resulted in the exclusion of 33 irrelevant citations, including 4 articles for reporting inappropriate subjects, 19 articles for being not accessible, 8 articles for not reporting outcomes of UPDRSIII or BBS, 1 article for not reporting results, and 1 article for repeating publication of the same study. Ultimately, 20 studies with a combined study population of 996 patients were included in this network meta-analysis (Figure 1), comparing the efficiency of 24-form, TCEP, 8-form YS, 8-form CS, and control group in improving movement disorders for treating PD. The characteristics of the selected articles are shown in Table 1.

**Table 1:** Basic characteristics of the included studies.

**Study**	**Sample size, *n* (T/C)**	**Age, year (mean** ±**SD)**		**Course of disease, year (mean** ±**SD)**		**TC intervention**	**Control group**	**Treatment sessions, *n***	**Total treatment course, week**	**Primary outcomes**
		**TC group**	**Control group**	**TC group**	**Control group**					
Gao et al. (2014)	37/39	69.54 ± 7.32	68.28 ± 8.53	9.15 ± 8.58	8.37 ± 8.24	24-form	NA	36	12	UPDRSIII, BBS
Ji et al. (2016)	16/16	56.06 ± 11.16	59.13 ± 11.22	2.09 ± 1.07	2.28 ± 1.18	8-form CS	NA	84	12	UPDRSIII, BBS
Lu (2017)	8/8	67.75 ± 6.84	68.20 ± 7.32	2.41 ± 0. 48	2.02 ± 0. 52	24-form	NA	40	8	UPDRSIII, BBS
Luan (2020)	13/13	64.08 ± 3.95	63.46 ± 4.33	NA	NA	TCEP	Stretching and walking	48	16	UPDRSIII, BBS
Wang et al. (2016)	40/40	67.6 ± 8.4	68.0 ± 8.5	NA	NA	24-form	NA	224	16	UPDRSIII, BBS
You and She (2020)	35/35	68.81 ± 5.02	68.49 ± 5.27	4.21 ± 0.24	4.17 ± 0.35	TCEP	Stretching and walking	48	24	UPDRSIII, BBS
Zhu et al. (2011)	19/19	63.35 ± 8.72	64.83 ± 9.29	2.72 ± 1.95	2.78 ± 2.29	24-form	Walking	20	4	UPDRSIII, BBS
Amano et al. (2013)	15/15	66 ± 11	66 ± 7	8 ± 5	D.5 ± 3	8-form YS	NA	32	16	UPDRSIII
Choi et al. (2013)	11/9	60.81 ± 7.6	65.54 ± 6.8	5.2 ± 2.7	5.2 ± 2.7	TCEP	NA	12	12	UPDRSIII
Deng (2020)	30/30	54.5 ± 1.2	54.4 ± 1.3	5.2 ± 1.0	5.1 ± 1.1	24-form	Na	60	12	BBS
Guan et al. (2016)	31/31	70.23 ± 4.24	69.71 ± 4.13	4.43 ± 3.17	4.28 ± 3.25	24-form	Na	48	12	BBS
Guan et al. (2017)	40/40	NA	NA	NA	NA	24-form	Walking	120	24	BBS
Guan et al. (2018)	40/40	69.46 ± 5.45	68.61 ± 6.22	1.52 ± 0.25	1.50 ± 0.32	24-form	NA	96	24	BBS
Li et al. (2017)	30/30	NA	NA	NA	NA	24-form	NA	48	12	BBS
Li et al. (2012)	65/65	A.68 ± 9	69 ± 9	A.8 ± 9	B.8 ± 9 C.6 ± 5	8-form YS	Stretching	48	24	UPDRSIII
Vergara-Diaz et al. (2018)	12/13	65.7 ± 3.86	62 ± 7.77	2.9 ± 2.38	2.9 ± 2.20	TCEP	NA	48	24	UPDRSIII
Wang (2015)	16/15	68.44 ± 4.604	68.20 ± 3.342	4.81 ± 2.287	4.93 ± 2.086	24-form	Walking	16	8	UPDRSIII
Xiao et al. (2021)	20/20	72.78 ± 2.63	72.58 ± 2.62	1.07 ± 0.17	1.08 ± 0.16	24-form	NA	96	24	BBS
Zhu (2017)	8/8	63.50 ± 6.78	61.50 ± 5.63	4.75 ± 2.43	3.88 ± 1.81	TCEP	Stretching and walking	48	24	UPDRSIII
Chen et al. (2016)	15/15	68.1 ± 8.2	68.2 ± 8.1	4.5 ± 0.7	4.6 ± 0.6	TCEP	NA	8	8	UPDRSIII, BBS

### Assessment of risk of bias and quality of studies

**Figure 2:**
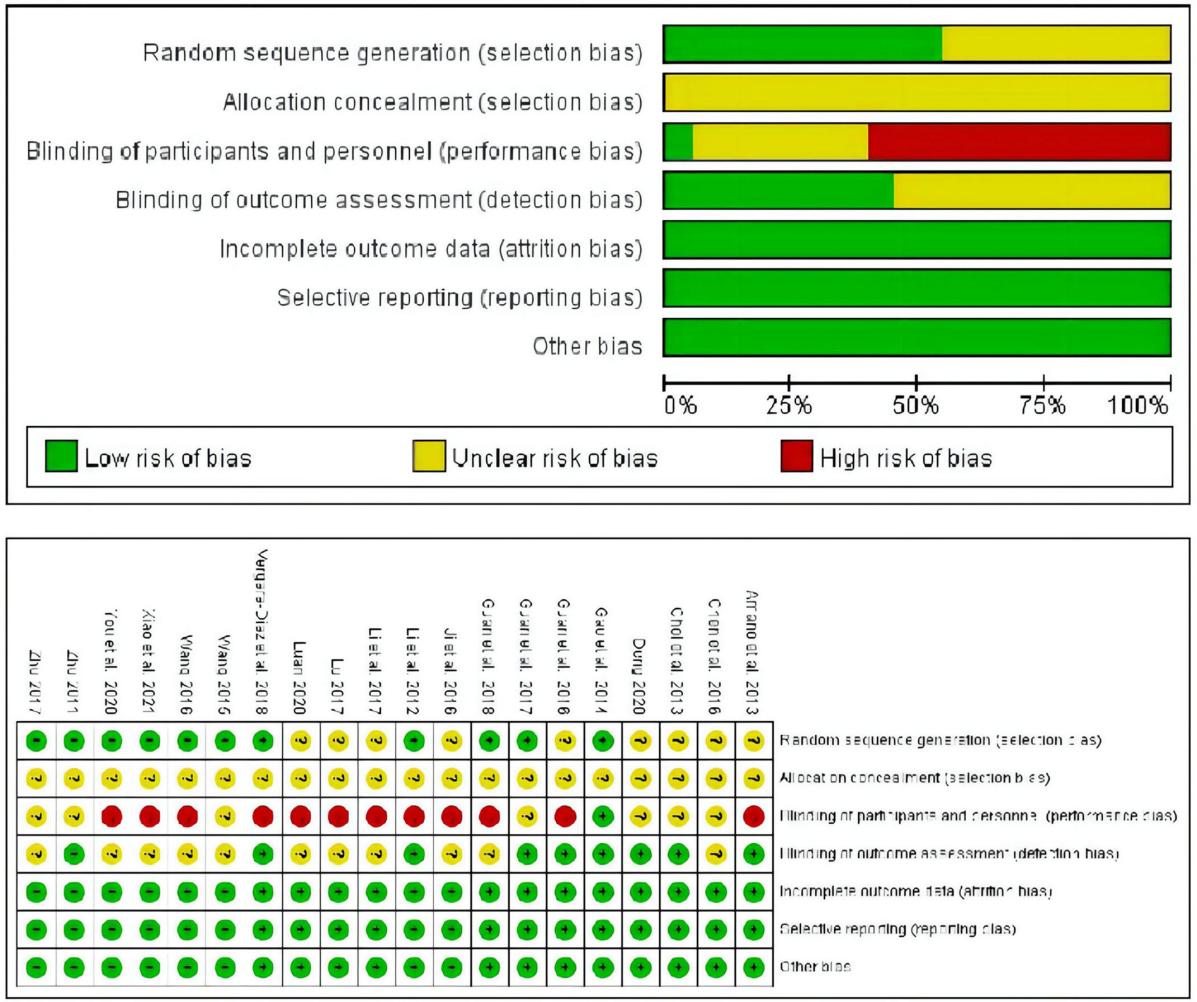
Quality assessment of included studies.

Following the Cochrane risk-of-bias tool, the quality of selected studies was assessed, and the results are shown in Figure 2. Of the included RCTs, eleven (55%) clearly described a random sequence generation process using a computer or a random number table. All trials had an uncertain risk of allocation concealment, and only one trial (5%) with a single-blind experiment had a low risk of performance bias. The blinding methods for outcome assessment were represented by nine trials (45%). All trials reported the expected outcome indicators (UPDRSIII scores or BBS scores), implying a lower risk of bias in reporting bias due to selective reporting. Meantime, the effect values (mean difference) of the missing data in all included trials were not sufficient to seriously affect the observed effect values, which resulted in a low risk of attrition bias for all of them.

### Results of a conventional meta-analysis

In terms of UPDRSIII, thirteen direct comparisons were constructed using a random effect model. Each pair of comparisons consisted of a control group and a specific type of Tai Chi group. As for the outcome, the 24-form (SMD = −1.272, 95% CI [−2.036, −0.508], *P* < 0.05, I^2^ > 50%) was statistically more efficient than the control group. TCEP (SMD = −0.839, 95% CI [−1.828, 0.151], *P* > 0.05, I^2^ > 50%), 8-form YS (SMD = −0.325, 95% CI [−1.362, 0.713], *P* > 0.05, I^2^ > 50%), and 8-form CS (SMD = −0.28, 95% CI [−0.97, 0.42], *P* > 0.05, I^2^ > 50%) did not show a significant difference between TC and control, with *P* > 0.05 reported.

In a classic pair-wise meta-analysis, fourteen direct comparisons including BBS scores were constructed. All interventions, except for the 24-form, used a random effect model while synthesizing RCTs with the same pair of interventions. For BBS outcome, 24-form (MD = 3.979, 95% CI [3.364, 4.595], *P* < 0.05, I^2^ <50%) and 8-form CS (MD = 1.25, 95% CI [0.52, 1.98], *P* < 0.05, I^2^ > 50%) were better than the control group. TCEP (MD = 5.00, 95% CI [2.07, 7.93], *P* > 0.05, I^2^ > 50%) did not show a significant difference between TC and control, with *P* > 0.05 reported. The detailed results of the conventional meta-analysis are presented in Supplementary Datasheet 2.

### Results of NMA

#### Unified Parkinson's disease rating scale III

**Figure 3:**
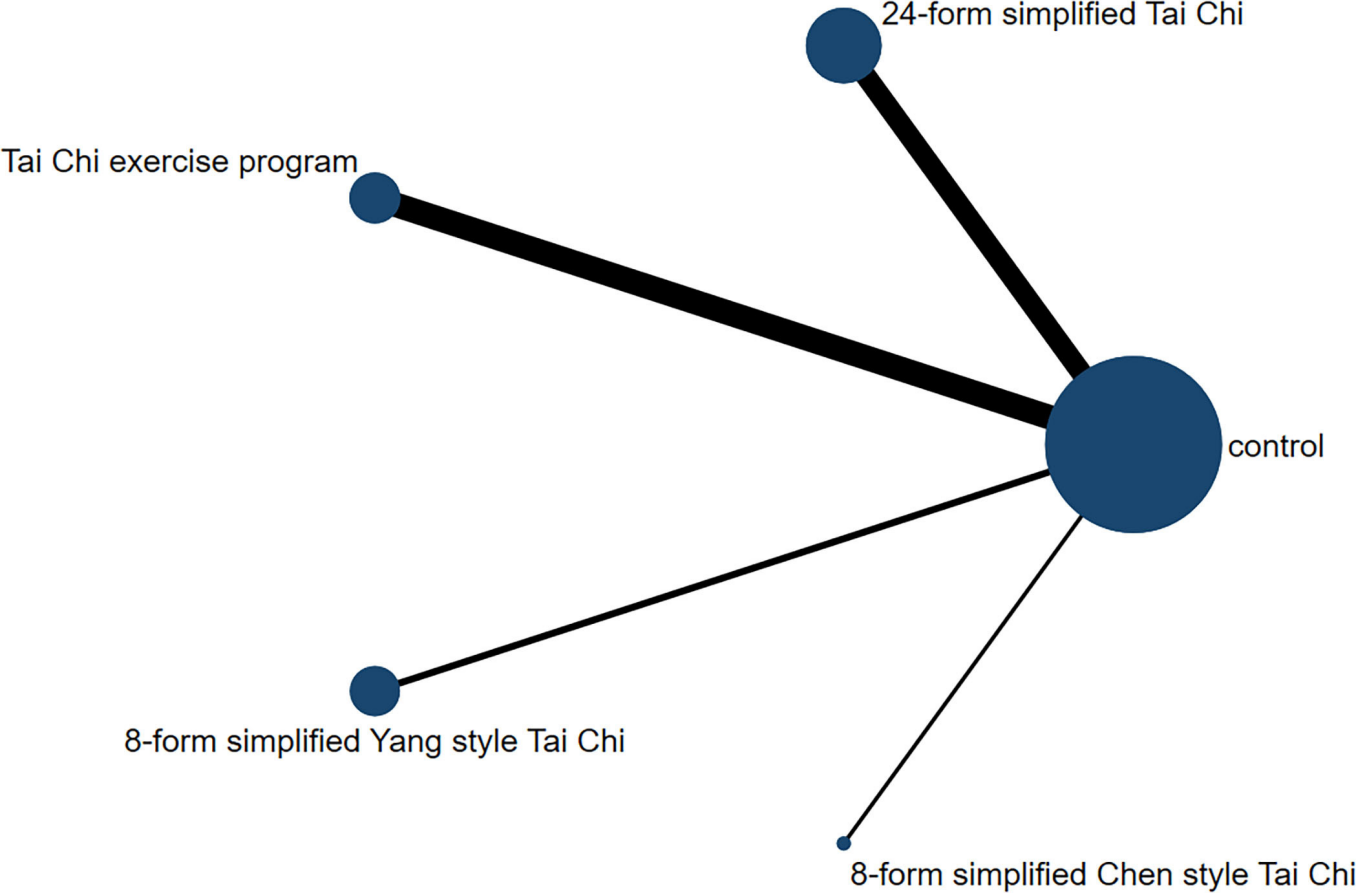
The network structure of the analyzed treatment comparisons for the outcome of UPDRSIII.

**Figure 4:**
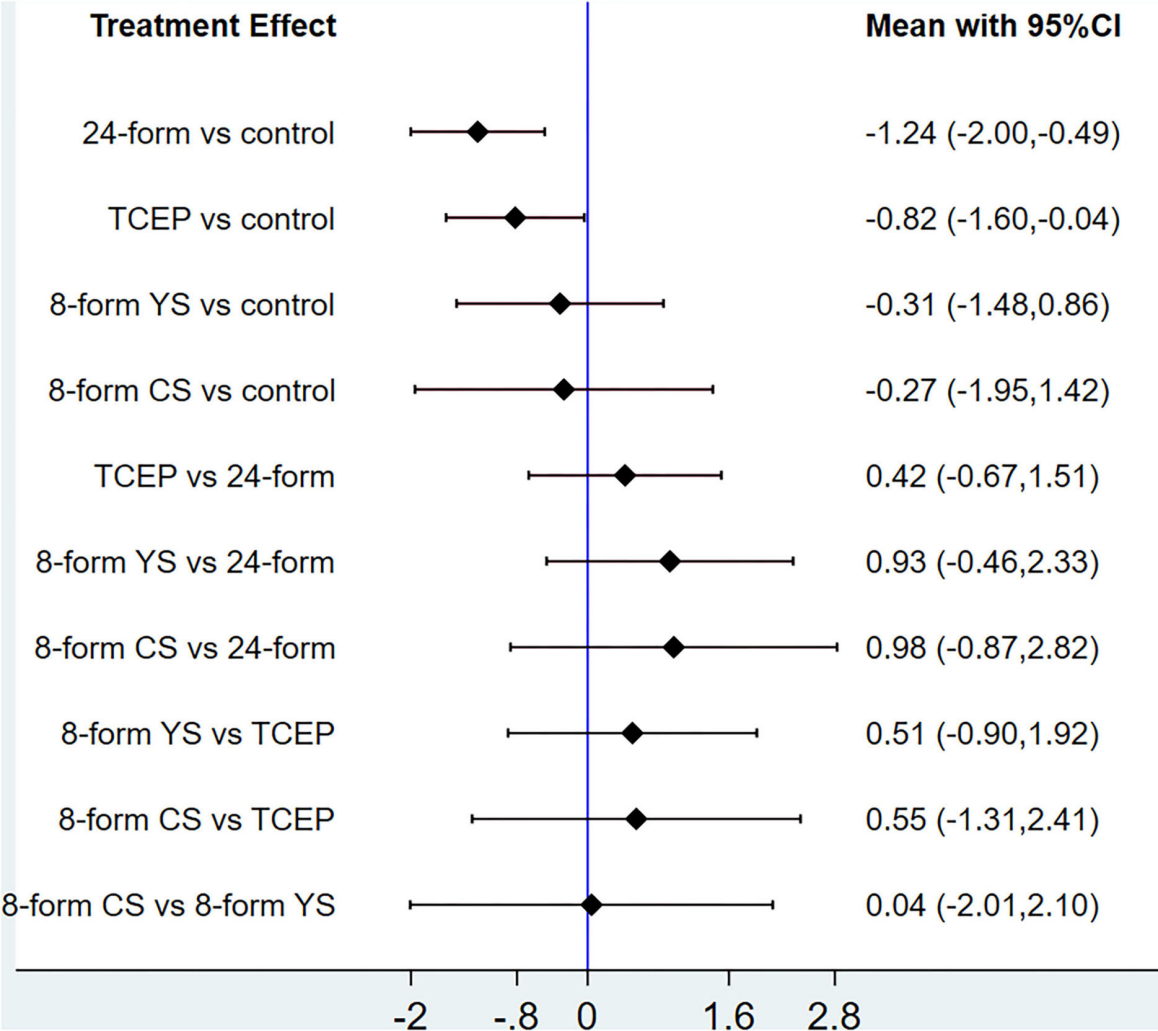
UPDRSIII: Interval plot comparing the effectiveness of the four treatments. 24-form: 24-form simplified Tai Chi; TCEP: Tai Chi exercise program; 8-form YS: 8-form simplified Yang style Tai Chi; 8-form CS: 8-form simplified Chen style Tai Chi.

**Figure 5:**
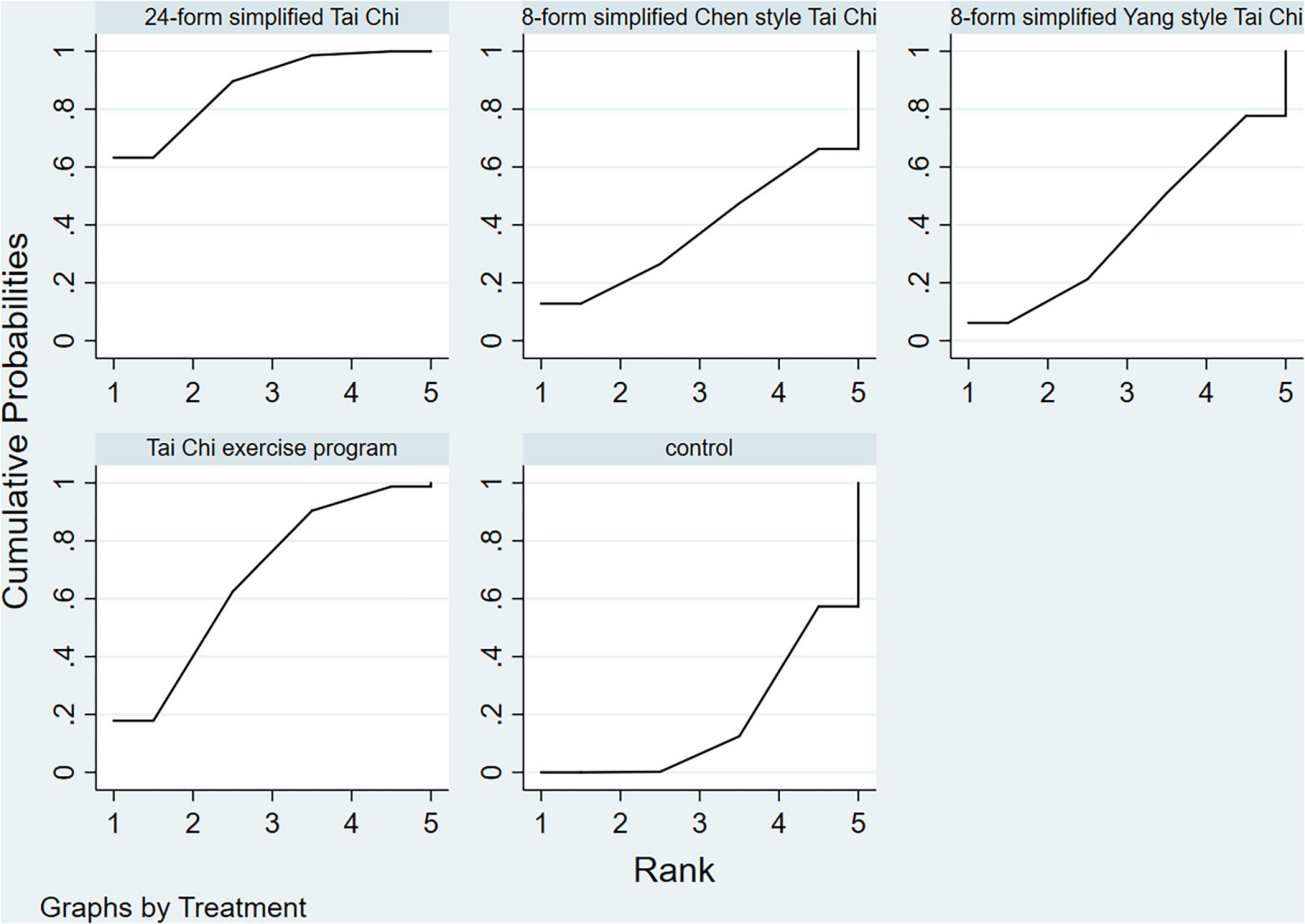
UPDRSIII rank probability.

**Figure 6:**
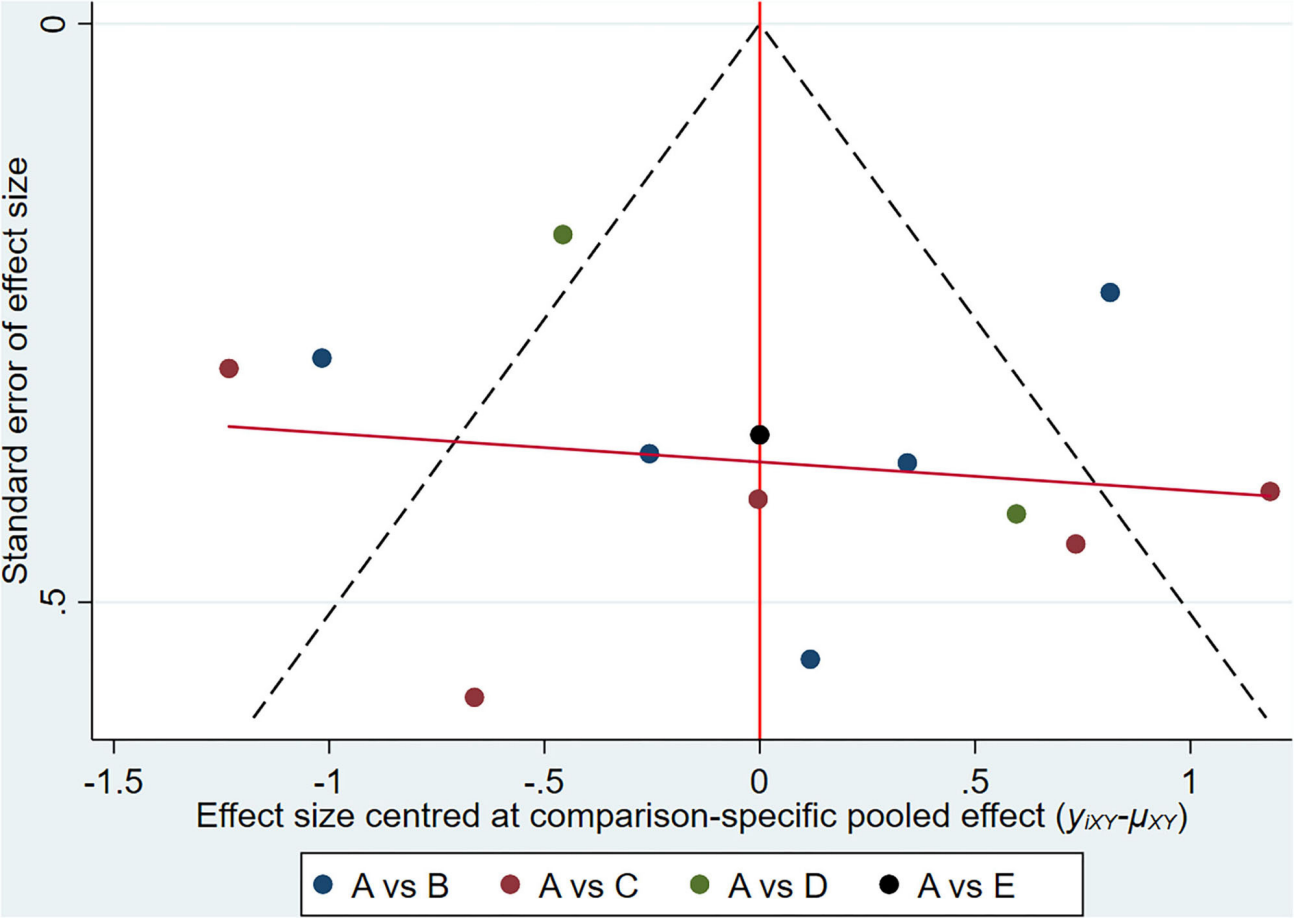
UPDRSIII: Funnel plot showing the publication bias of the included randomized controlled trials. (A) control group; (B) 24-form simplified Tai Chi; (C) Tai Chi exercise program; (D) 8-form simplified Yang style Tai Chi; (E) 8-form simplified Chen style Tai Chi.

In relation to the UPDRSIII score, 13 trials and 5 treatments constituted a network diagram, 5 arms of 24-form, 5 arms of TCEP, 2 arms of 8-form YS, and 1 arm of 8-form CS (presented in Figure 3). Due to the lack of loops in the network graph, we directly used a consistency model to compare these 5 interventions. The interval plot showed that 24-form (SMD: −1.24; 95% CI: −2.00, −0.49) and TCEP (SMD: −0.82; 95% CI: −1.60, −0.04) were better than the control group. While 8-form YS (SMD: −0.31; 95% CI: −1.48, 0.86) and 8-form CS (SMD: −0.27; 95% CI: −1.95, 1.42) did not show a significant difference between TC and control (Figure 4). The ranking probability of UPDRSIII was constituted by the SUCRA, and the results were as follows: 24-form > TCEP > 8-form YS > 8-form CS > control (Figure 5). Regarding the funnel plot, it revealed an asymmetric with a fitting line almost vertical to the zero line, suggesting that the study might have a small sample effect or a publication bias (Figure 6). Later, Egger's test suggested that there was no publication bias (*P* = 0.262 > 0.1).

#### Berg balance scale

**Figure 7:**
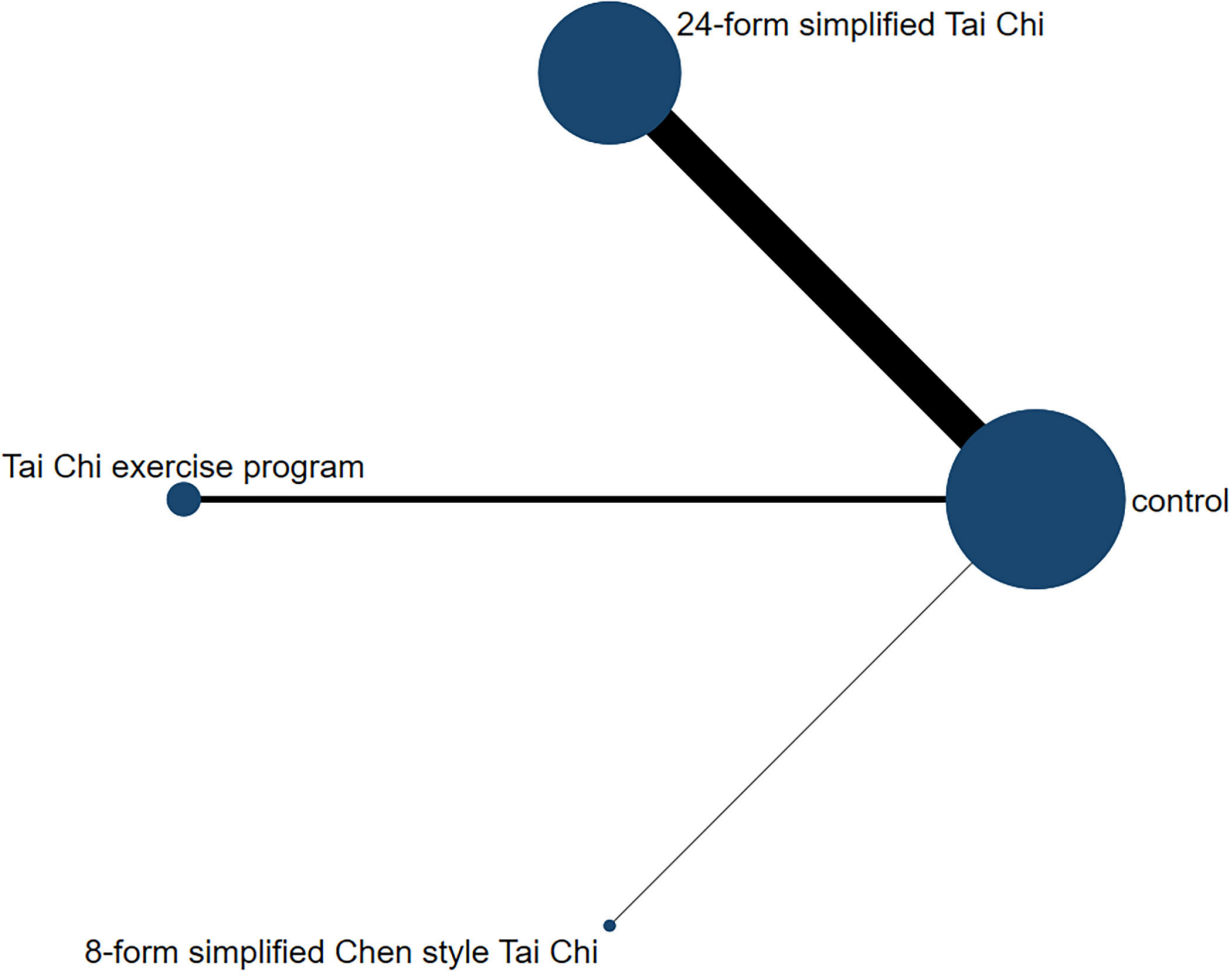
The network structure of the analyzed treatment comparisons for the outcome of BBS.

**Figure 8:**
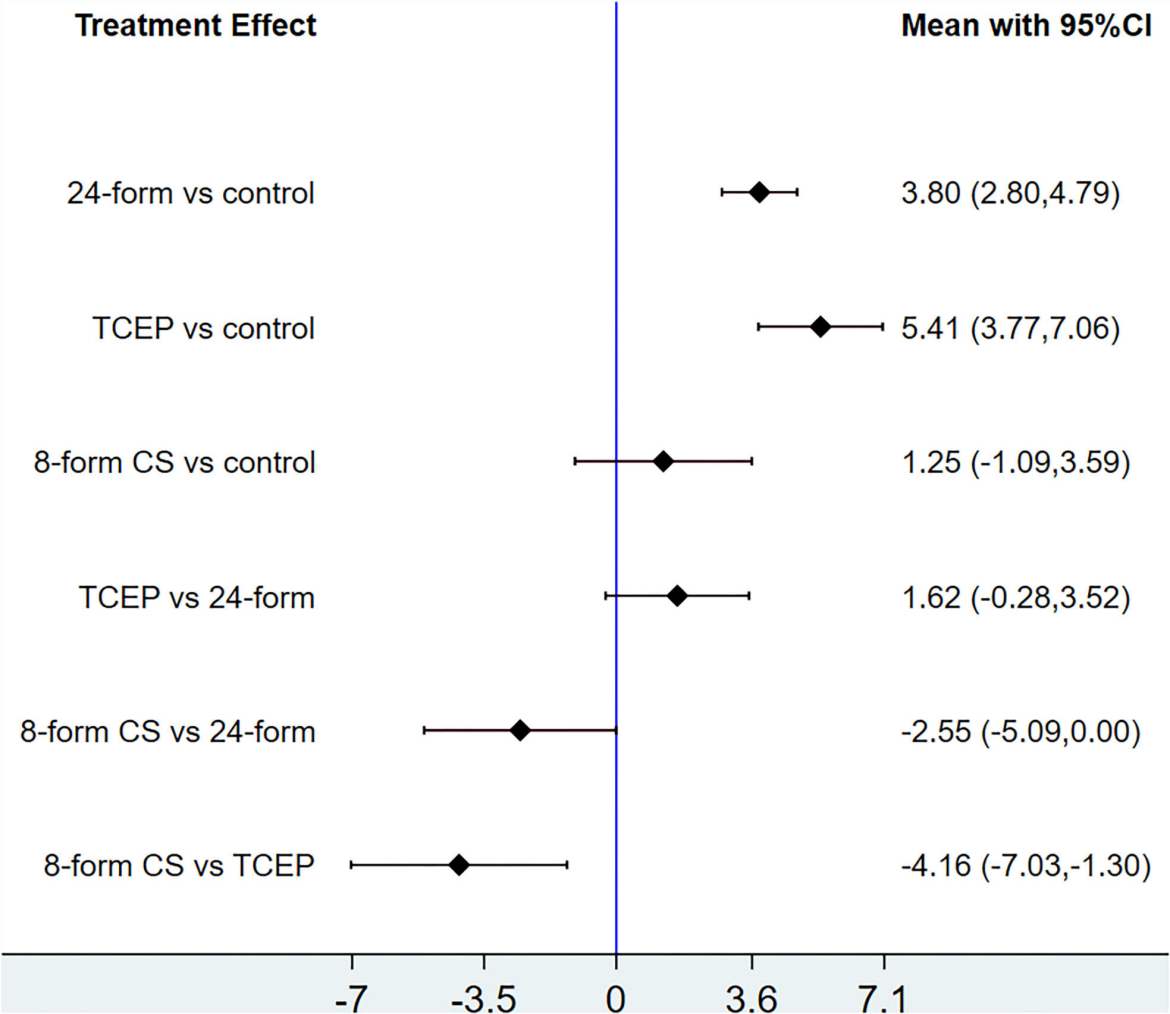
BBS: Interval plot comparing the effectiveness of the four treatments. 24-form: 24-form simplified Tai Chi; TCEP: Tai Chi exercise program; 8-form CS: 8-form simplified Chen style Tai Chi.

**Figure 9:**
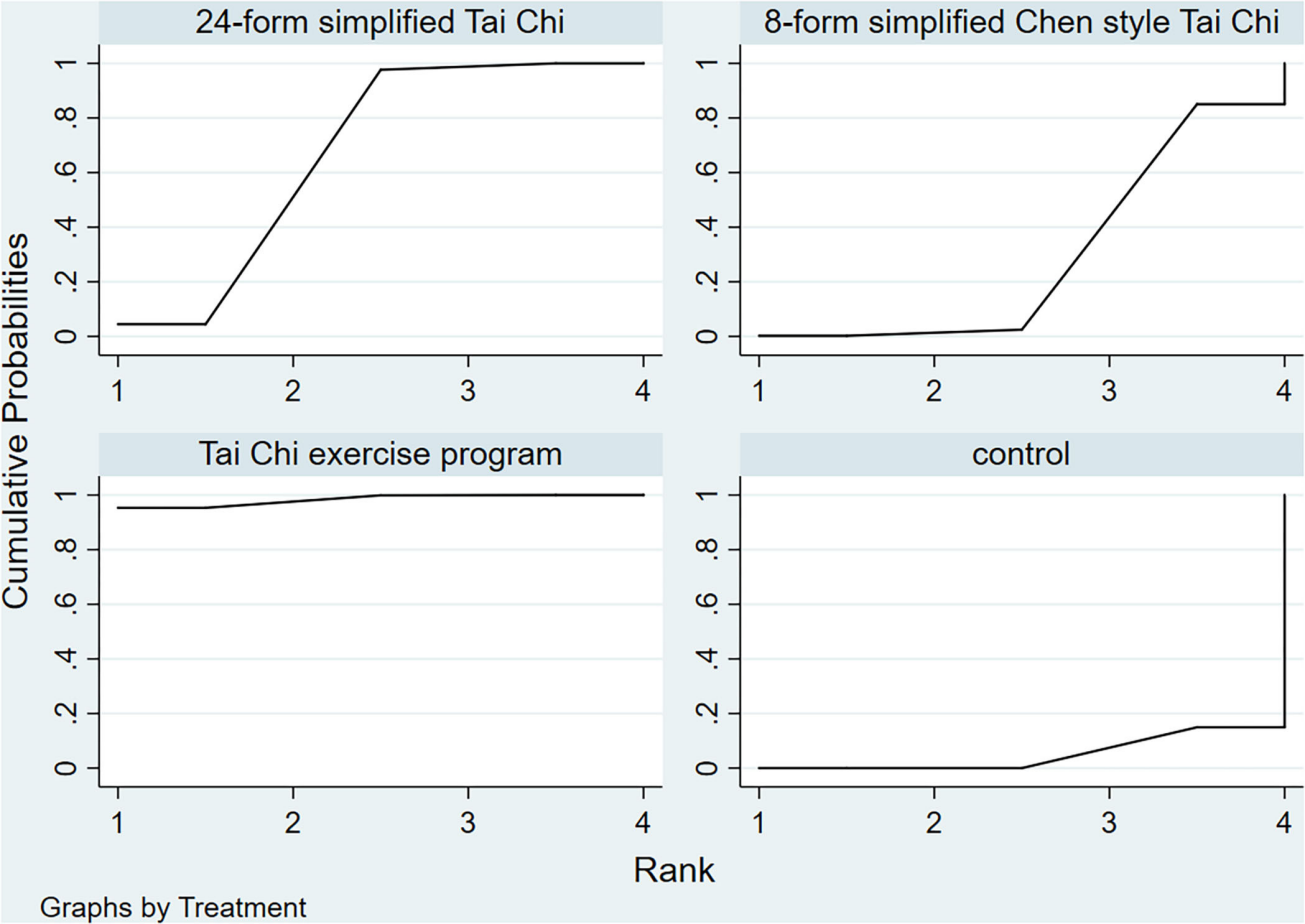
BBS rank probability.

**Figure 10:**
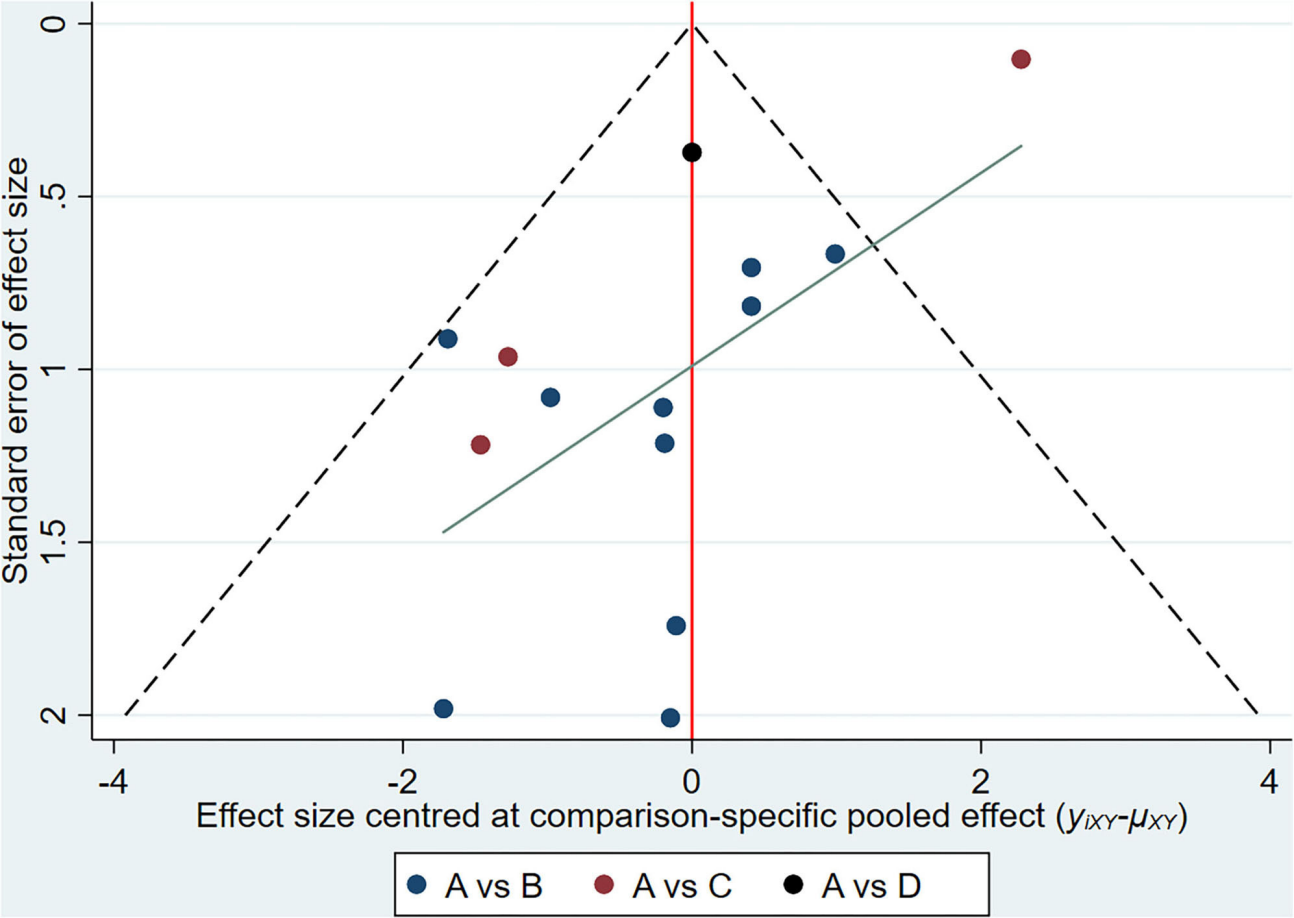
BBS: Funnel plot showing the publication bias of the included randomized controlled trials. (A) control group; (B) 24-form simplified Tai Chi; (C) Tai Chi exercise program; (D) 8-form simplified Chen style Tai Chi.

Figure 7 shows the relationship among the 4 treatments in a network diagram, 10 RCTs comparing 24-form with the control group, 3 RCTs comparing TCEP with the control group, and 1 RCT comparing 8-form CS with the control group. Since there were no loops in the network graph, comparisons of the five interventions were directly constituted by a consistency model. In terms of 24-form (MD: 3.80 95% CI: 2.80, 4.79) and TCEP (MD: 5.41; 95% CI: 3.77, 7.06) achieved better than the control group. In addition, 8-form CS (MD: 1.25; 95% CI: −1.09, 3.59) did not show a significant difference between TC and control (presented in Figure 8). The SUCRA showed the ranking probability of BBS as follows: TCEP > 24-form > 8-form CS > control (Figure 9). The funnel plot revealed asymmetry with a fitting line almost vertical to the zero line, which suggested that the study might have a small sample effect or a publication bias (Figure 10). Later, Egger's test suggested that there was no publication bias (*P* = 0.407 > 0.1).

## Discussion

Tai Chi can show long-term improvements in the balance and motor ability of elderly patients with PD to a certain extent (Huang et al., 2015). We have studied current articles on improving exercise capacity in elderly patients with PD. However, it was found that there were few reports of differences in outcomes associated with different types of Tai Chi.

To assess the best type of Tai Chi for the treatment of PD, we performed a network meta-analysis based on data from 20 RCTs (combined *n* = 996). All of them compared different types of Tai Chi vs. the control group. Motor control and balance ability are the therapeutic goals of elderly patients with PD. Therefore, in this study, we analyzed UPDRSIII scores and BBS scores for treatment durations ranging from 4 to 24 weeks. In the network meta-analysis, the SUCRA values of UPDRSIII were sorted from high to low: 24-form > TCEP > 8-form YS > 8-form CS > control. The ranking probability of BBS was as follows: TCEP > 24-form > 8-form CS > control.

For the UPDRS-III outcome, 24-form showed better efficacy than other types, followed by TCEP. Only the 24-form's included RCTs showed statistically significant differences in efficacy when compared with the control groups, while the other three types of Tai Chi's included RCTs did not. This indicated that 24-form Tai Chi can better improve the motor function of patients with PD. The diagonal movement in the 24-form is very prominent (Zhu et al., 2011), which uses the facilitation technique to improve cortical excitability (Zhu et al., 2008). Thanks to the feedforward and feedback control mechanisms, these movements allow for better motor control and could overcome problems such as difficulty in gait initiation when exercising limbs (Gallagher, 2003). The whole exercise process of 24-form is performed alternately by one leg or two legs to support one's body weight. Therefore, the quadriceps femoral muscle can be relaxed and tense alternately (Yang et al., 2009). During exercise, the center of gravity of the human body constantly moves between the two feet (Wang, 2015). Increasing the angle of hip flexion and knee flexion could keep the center of gravity of the body in a lower position and promote the completion of antigravity muscle strength training of the lower limbs (Zhu et al., 2011). These movements can strengthen the elderly lower limb muscle strength and muscular endurance, and improve coordination and balance (Low et al., 2009; Lu, 2017). The TCEP was the second better one. It was because the TCEP was mainly adapted from traditional Tai Chi movements and was formulated for treating different PD motor symptoms. Most of them involved diagonal movements and weight shifting training. Therefore, the UPDRSIII outcome of TCEP was also ranked very high.

For BBS outcome, TCEP showed the best efficacy among the 4 types of Tai Chi, followed by the 24-form. However, the efficacy of traditional Tai Chi is not as good as that of TCEP and 24-form. We believe that the reason may be related to the fixed movement of traditional Tai Chi, which is similar to the opinion of some scholars (You and She, 2020). In our study, the action selection of TCEP is diverse, and different protocols may cause different results. The 2 included RCTs (Luan, 2020; You and She, 2020) aimed at improving balance ability and selected the movements of weight shifting and balance training to reduce the burden of balance and gait. Among the included RCTs, the trunk movements selected by most of the Tai Chi exercise programs (Chen et al., 2016; Vergara-Diaz et al., 2018) can increase the interarticular pressure, improve the patient's proprioceptive input, and promote the patient's postural stability (Tsang and Hui-Chan, 2003).

From the above, we can conclude that, as a moderate-intensity aerobic exercise, Tai Chi is beneficial with regard to muscle strength, balance, physical coordination, and postural stability for people with PD. However, part of the included literature mentioned stretching or walking training in the control group (Zhu et al., 2011; Li et al., 2012; Wang, 2015; Guan et al., 2017; Zhu, 2017; Luan, 2020; You and She, 2020). By comparison, we found that Tai Chi exercise was more dominant in improving motor function. In addition, because of its slow speed, simple, and interesting movements, Tai Chi is suitable for promotion in a crowd and could be highly accepted by patients. The findings of our study have guiding significance for the type selection of clinical exercise prescription.

Our study has some other potential limitations. First, significant heterogeneity was observed in the conventional meta-analysis. This is likely attributable to differences in practice frequency, treatment sessions, and daily living habits of patients. Besides, different interventions were taken in the control group, which might also be one of the reasons. Fortunately, as the obtained network diagrams were short of closed loops, no inconsistency or heterogeneity was reported in the network meta-analysis. However, there is a probability that the effectiveness of treatments in some included studies might have been influenced, and this might affect the results of our study. Second, the included reports used non-pharmaceutical therapies, which could not be blinded to participants. However, this study included trials using single-blind methodologies to reduce the potential for any bias. Finally, we have excluded some trials due to selection criteria for inclusion, which may influence the strength of the results reported by the NMA. Considering the above limitations, our findings should be interpreted with caution.

Further investigation is required to evaluate other TC interventions not included in this article. For example, Ai Chi is a different type of Tai Chi, including exercise in water. A study compared the changes in UPDRS III scores and BBS scores between the Ai Chi group and the land-based exercise group, finding that the Ai Chi group showed better improvement in motor function of patients with PD. Besides, the exercise for the proprioceptive system in water could also improve postural control (Kurt et al., 2017). Considering the inclusion and exclusion criteria, our article did not include this trial, but the special type of Tai Chi shown in these RCTs, Ai Chi, could be provided as a choice for exercise prescription for treatment of the elderly PD.

Due to the complications of pharmaceutical therapy in patients with PD, studies on non-pharmaceutical therapy have been rapidly developed and advanced, including resistance exercise, aerobic exercise, stretching, and walking. Tai Chi as aerobic exercise has been proven to be an effective treatment, and there are more and more Tai Chi exercise programs specially formulated for Parkinson's patients to improve the efficacy and effectiveness of this therapy. Clinically, it is recommended to make a reasonable exercise prescription according to the patient's physical condition and treatment goals, selecting appropriate exercise frequency, exercise intensity, exercise time, and so on. According to our study, for those who want to improve their motor function, it is recommended to practice 24-form simplified Tai Chi, and a Tai Chi exercise program is recommended for those who want to improve their balance. If the patients want to improve both motor function and balance, TCEP is the main recommendation for them.

## Data availability statement

The raw data supporting the conclusions of this article will be made available by the authors, without undue reservation.

## Author contributions

HL and XZ designed the study. HL, KT, and ZM contributed to the literature research, the study selection, and the data extraction. XZ checked the data and information checking and contributed to conceptualization, methodology, supervision, funding acquisition, and project administration. JW and HL performed the quality assessment. HL and KL analyzed the data. BM, YC, QY, and HJ methodology, formal analysis, and software. All authors contributed to the article and approved the submitted version.

## Funding

This study was funded by a grant from the National Natural Science Foundation of China (No. 82105034).

## Conflict of interest

The authors declare that the research was conducted in the absence of any commercial or financial relationships that could be construed as a potential conflict of interest.

## Publisher's note

All claims expressed in this article are solely those of the authors and do not necessarily represent those of their affiliated organizations, or those of the publisher, the editors and the reviewers. Any product that may be evaluated in this article, or claim that may be made by its manufacturer, is not guaranteed or endorsed by the publisher.
