# Supplementary material for: The effects of different types of Tai Chi exercises on motor function in patients with Parkinson's disease: A network meta-analysis
Source: Front Aging Neurosci. 2022 Aug 29;14:936027. doi: 10.3389/fnagi.2022.936027 (PMC9465240; doi:10.3389/fnagi.2022.936027)
Supplement: Supplementary file 1 [file Data_Sheet_1.docx]

| **Supplementary Datasheet 1.** Search strategy. | |
| --- | --- |
| Source: PubMed; searched on January 14, 2022 | |
| Search | Query |
| #1 | (Tai Ji[MeSH Terms]) OR (Tai-ji[Title/Abstract]) OR (Tai Chi[Title/Abstract]) OR (Chi, Tai[Title/Abstract]) OR (Tai Ji Quan[Title/Abstract]) OR (Ji Quan, Tai[Title/Abstract]) OR (Quan, Tai Ji[Title/Abstract]) OR (Taiji[Title/Abstract]) OR (Taijiquan[Title/Abstract]) OR (T'ai Chi[Title/Abstract]) OR (Tai Chi Chuan[Title/Abstract]) OR (Mind-Body Therapies[Title/Abstract]) OR (Mind Body Therapies[Title/Abstract]) OR (Mind-Body Therapy[Title/Abstract]) OR (Therapies, Mind-Body[Title/Abstract]) OR (Therapy, Mind-Body[Title/Abstract]) OR (Mind-Body Medicine[Title/Abstract]) OR (Mind Body Medicine[Title/Abstract]) OR (Exercise Movement Techniques[Title/Abstract]) OR (Movement Techniques, Exercise[Title/Abstract]) OR (Exercise Movement Technics[Title/Abstract]) OR (Pilates-Based Exercises[Title/Abstract]) OR (Exercises, Pilates-Based[Title/Abstract]) OR (Pilates Based Exercises[Title/Abstract]) OR (Pilates Training[Title/Abstract]) OR (Training, Pilates[Title/Abstract]) OR (Martial Arts[Title/Abstract]) OR (Arts, Martial[Title/Abstract]) OR (Hap Ki Do[Title/Abstract]) OR (Judo[Title/Abstract]) OR (Karate[Title/Abstract]) OR (Jujitsu[Title/Abstract]) OR (Tae Kwon Do[Title/Abstract]) OR (Aikido[Title/Abstract]) OR (Wushu[Title/Abstract]) OR (Kung Fu[Title/Abstract]) OR (Gong Fu[Title/Abstract]) OR (Fu, Gong[Title/Abstract]) OR (Gongfu[Title/Abstract]) |
| #2 | (Parkinson Disease[MeSH Terms]) OR (Idiopathic Parkinson's Disease[Title/Abstract]) OR (Lewy Body Parkinson's Disease[Title/Abstract]) OR (Parkinson's Disease, Idiopathic[Title/Abstract]) OR (Parkinson's Disease, Lewy Body[Title/Abstract]) OR (Parkinson Disease, Idiopathic[Title/Abstract]) OR (Parkinson's Disease[Title/Abstract]) OR (Idiopathic Parkinson Disease[Title/Abstract]) OR (Lewy Body Parkinson Disease[Title/Abstract]) OR (Primary Parkinsonism[Title/Abstract]) OR (Parkinsonism, Primary[Title/Abstract]) OR (Paralysis Agitans[Title/Abstract]) OR (Parkinsonian Disorders[Title/Abstract]) OR (Parkinsonian Syndrome[Title/Abstract]) OR (Parkinsonian Syndromes[Title/Abstract]) OR (Parkinsonian Diseases[Title/Abstract]) OR (Parkinsonism[Title/Abstract]) OR (Autosomal Dominant Parkinsonism[Title/Abstract]) OR (Dominant Parkinsonism, Autosoma[Title/Abstract]) OR (Parkinsonism, Autosomal Dominant[Title/Abstract]) OR (Autosomal Recessive Juvenile Parkinson Disease[Title/Abstract]) OR (Juvenile Parkinson Disease, Autosomal Recessive[Title/Abstract]) OR (Juvenile Parkinsonism, Autosomal Recessive[Title/Abstract]) OR (Parkinsonism, Juvenile, Autosomal Recessive[Title/Abstract]) OR (Familial Parkinson Disease, Autosomal Recessive[Title/Abstract]) OR (Autosomal Recessive Juvenile Parkinsonism[Title/Abstract]) OR (Parkinson Disease 2, Autosomal Recessive Juvenile[Title/Abstract]) OR (Parkinson Disease, Juvenile, Autosomal Recessive[Title/Abstract]) OR (Parkinson Disease 2[Title/Abstract]) OR (Parkinson Disease Autosomal Recessive, Early Onset[Title/Abstract]) OR (Chromosome 6-Linked Autosomal Recessive Parkinsonism[Title/Abstract]) OR (Chromosome 6 Linked Autosomal Recessive Parkinsonism[Title/Abstract]) OR (Parkinson Disease, Familial, Autosomal Recessive[Title/Abstract]) OR (Ramsay Hunt Paralysis Syndrome[Title/Abstract]) OR (Familial Juvenile Parkinsonism[Title/Abstract]) OR (Juvenile Parkinsonism, Familial[Title/Abstract]) OR (Parkinsonism, Familial Juvenile[Title/Abstract]) OR (Parkinsonism, Experimental[Title/Abstract]) OR (Experimental Parkinsonisms[Title/Abstract]) OR (Parkinsonisms, Experimental[Title/Abstract]) OR (Parkinson Disease, Experimental[Title/Abstract]) OR (Experimental Parkinsonism, MPTP-Induced[Title/Abstract]) OR (Experimental Parkinsonism, MPTP Induced[Title/Abstract]) OR (Parkinsonism, MPTP-Induced Experimental[Title/Abstract]) OR (MPTP-Induced Experimental Parkinsonism[Title/Abstract]) OR (MPTP Induced Experimental Parkinsonism[Title/Abstract]) OR (Experimental Parkinson Disease[Title/Abstract]) OR (Diseases, Experimental Parkinson[Title/Abstract]) OR (Experimental Parkinson Diseases[Title/Abstract]) OR (Parkinson Diseases, Experimental[Title/Abstract]) OR (Experimental Parkinsonism[Title/Abstract]) OR (Parkinsonism, Juvenile[Title/Abstract]) OR (Juvenile Parkinsonism[Title/Abstract]) OR (Juvenile Parkinsonisms[Title/Abstract]) OR (Parkinsonisms, Juvenile[Title/Abstract]) OR (Parkinson Disease, Juvenile[Title/Abstract]) OR (Juvenile Parkinson Disease[Title/Abstract]) OR (Autosomal Dominant Juvenile Parkinson Disease[Title/Abstract]) OR (Autosomal Dominant Juvenile Parkinsonism[Title/Abstract]) OR (Parkinsonism, Juvenile, Autosomal Dominant[Title/Abstract]) OR (Parkinson Disease, Autosomal Dominant. Juvenile[Title/Abstract]) OR (Parkinson Disease, Juvenile, Autosomal Dominant[Title/Abstract]) OR (Juvenile Parkinsonism, Autosomal Dominant[Title/Abstract]) OR (Juvenile Parkinson Disease, Autosomal Dominant[Title/Abstract]) OR (Autosomal Recessive Parkinsonism[Title/Abstract]) OR (Parkinsonism, Autosomal Recessive[Title/Abstract]) OR (Recessive Parkinsonism, Autosomal[Title/Abstract]) OR (Synucleinopathies[Title/Abstract]) OR (Synucleinopathy[Title/Abstract]) OR (a-Synucleinopathies[Title/Abstract]) OR (a-Synucleinopathy[Title/Abstract]) OR (alpha Synuclein Pathology[Title/Abstract]) OR (alpha Synuclein Pathologies[Title/Abstract]) OR (alpha-Synucleinopathies[Title/Abstract]) OR (alpha Synucleinopathies[Title/Abstract]) OR (alpha-Synucleinopathy[Title/Abstract]) OR (Parkinsonism, Early Onset, Diurnal Fluctuation[Title/Abstract]) OR (Parkinsonism, Early-Onset, Diurnal Fluctuation[Title/Abstract]) |
| #3 | ("Randomized Controlled Trial" [Publication Type]) OR (randomized[Title/Abstract]) OR (placebo[Title/Abstract]) |
| #4 | #1 AND #2 AND #3 |
