# Supplementary material for: The effects of different types of Tai Chi exercises on motor function in patients with Parkinson's disease: A network meta-analysis
Source: Front Aging Neurosci. 2022 Aug 29;14:936027. doi: 10.3389/fnagi.2022.936027 (PMC9465240; doi:10.3389/fnagi.2022.936027)
Supplement: Supplementary file 2 [file Data_Sheet_2.docx]

**
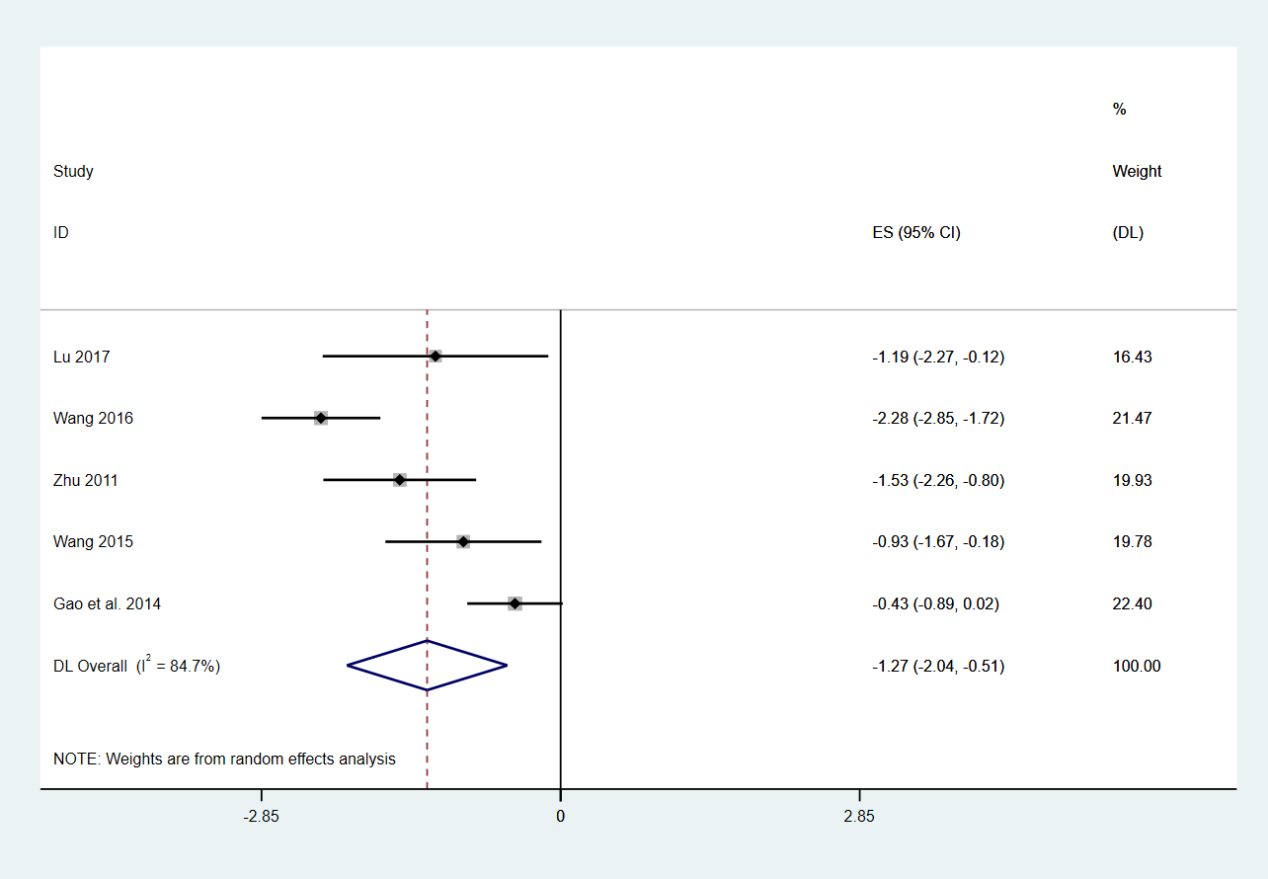
**

**Supplementary Datasheet 2.1.** Results of conventional meta-analysis, UPDRSⅢ, 24-form


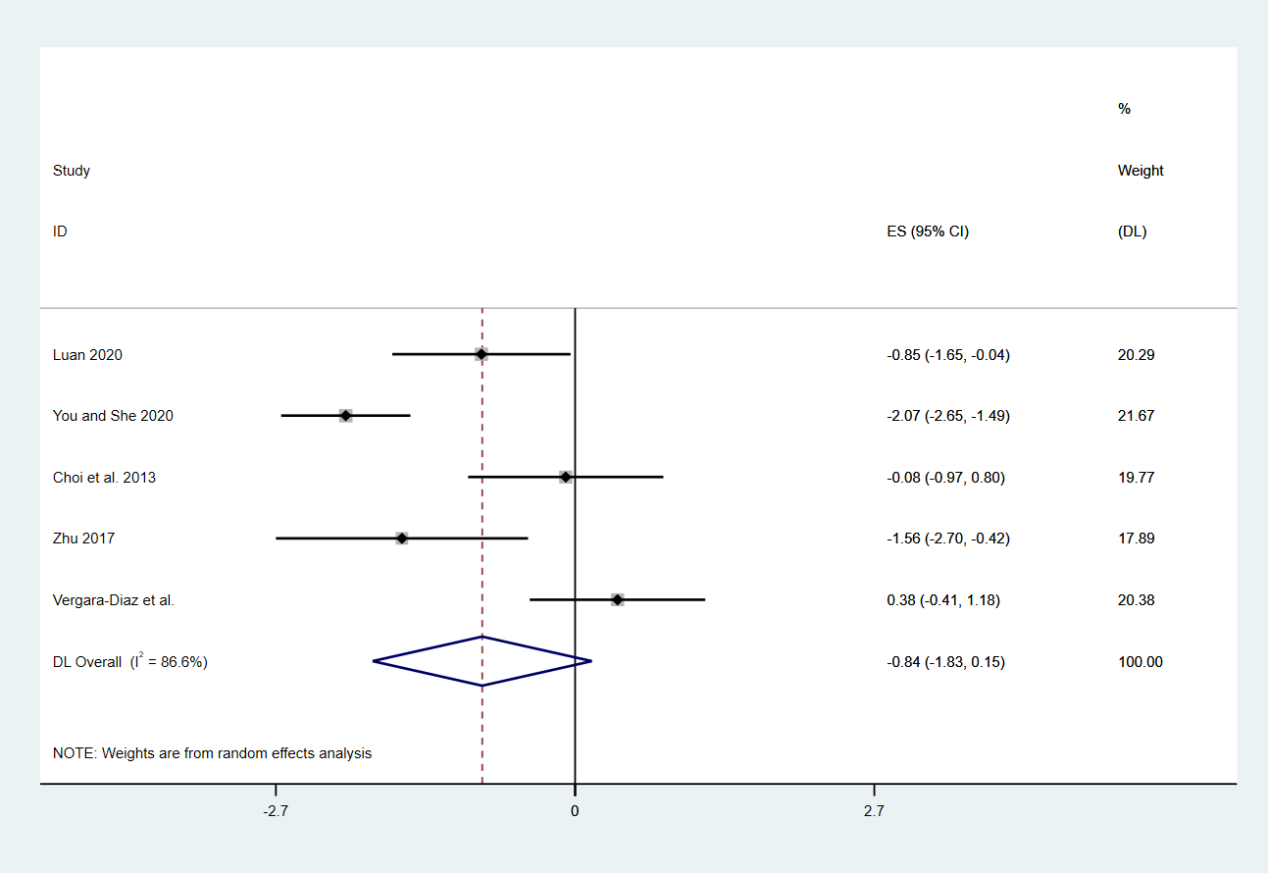


**Supplementary Datasheet 2.2.** Results of conventional meta-analysis, UPDRSⅢ, TCEP


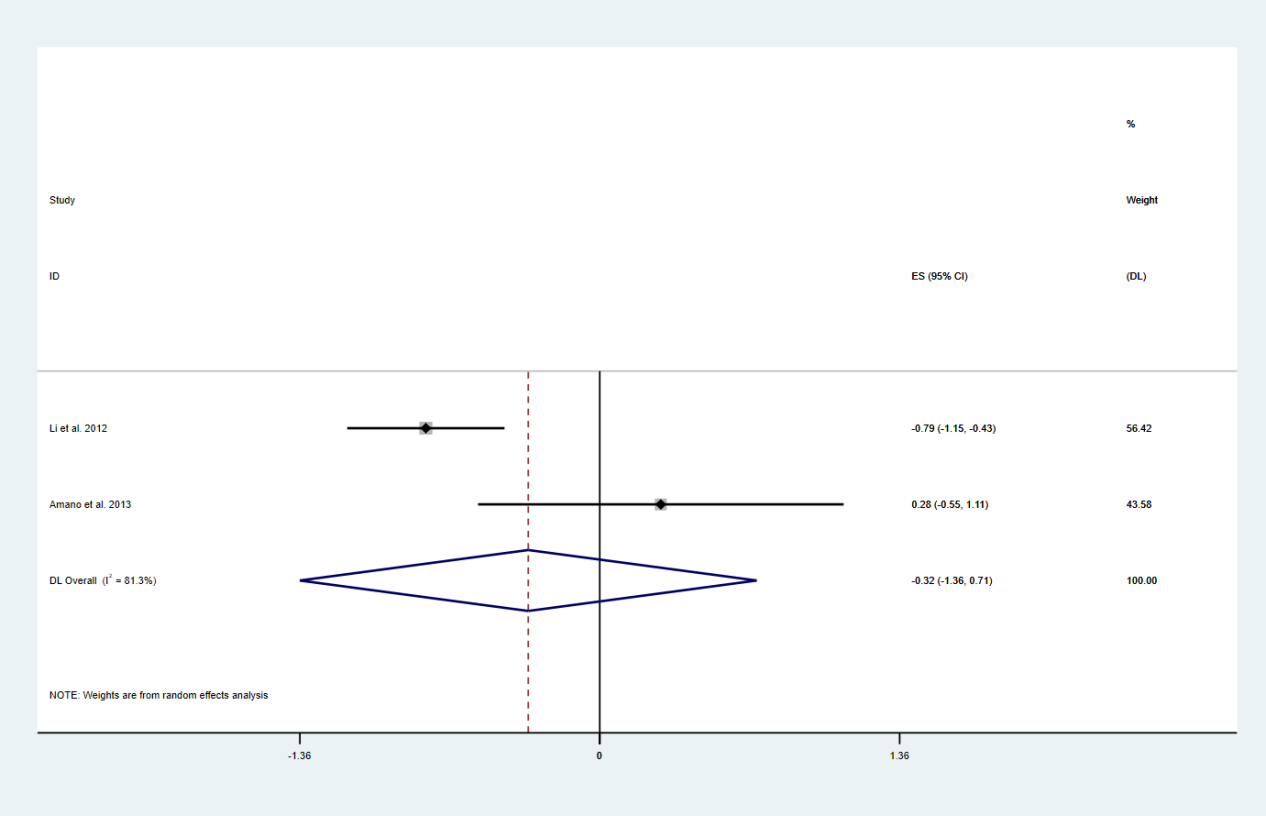


**Supplementary Datasheet 2.3.** Results of conventional meta-analysis, UPDRSⅢ, 8-form YS


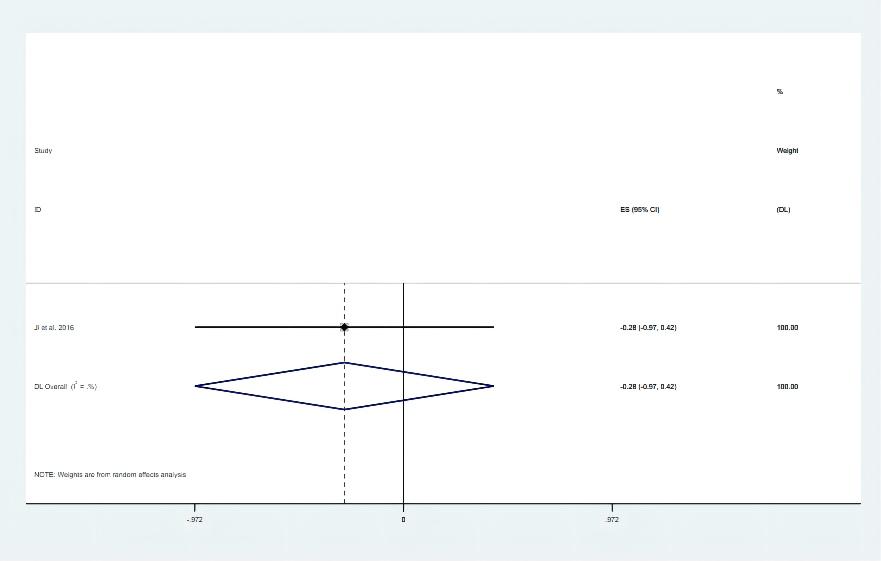


**Supplementary Datasheet 2.4.** Results of conventional meta-analysis, UPDRSⅢ, 8-form CS


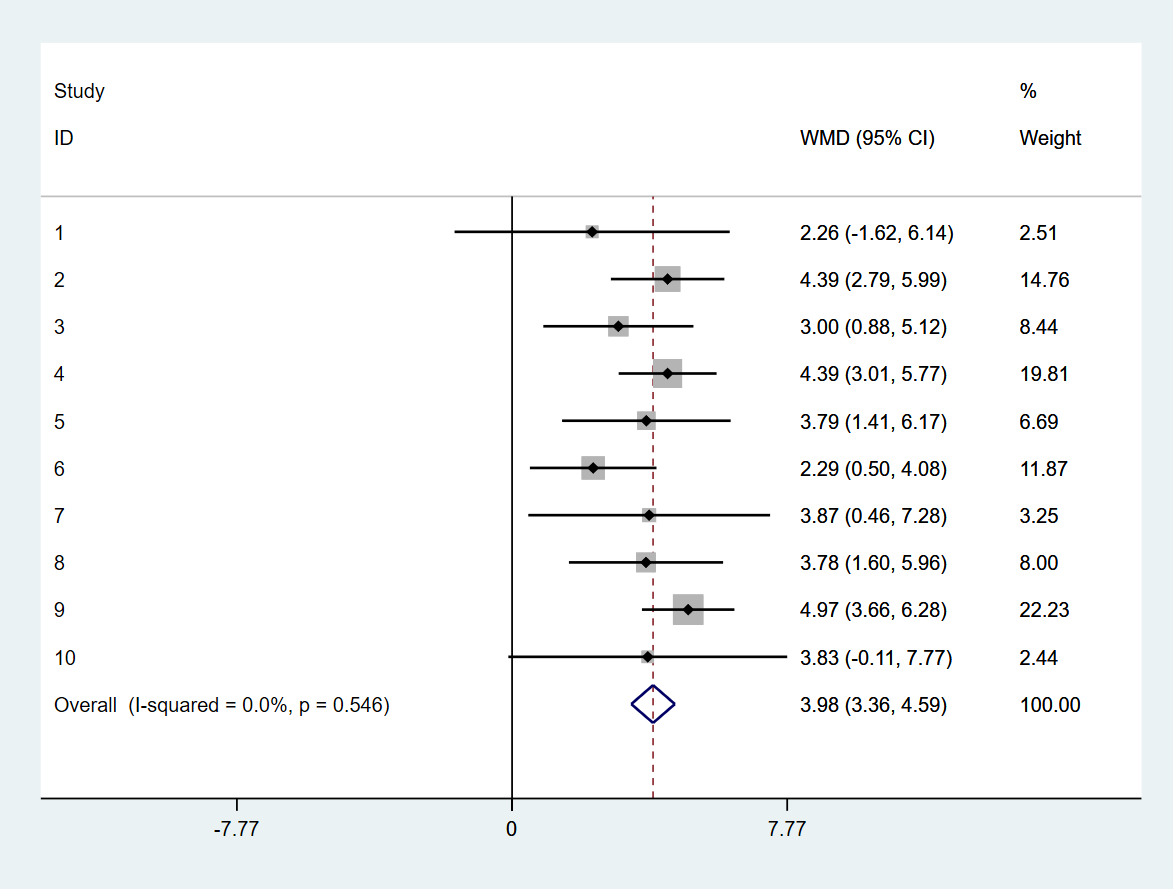


**Supplementary Datasheet 2.5.** Results of conventional meta-analysis，BBS, 24-form


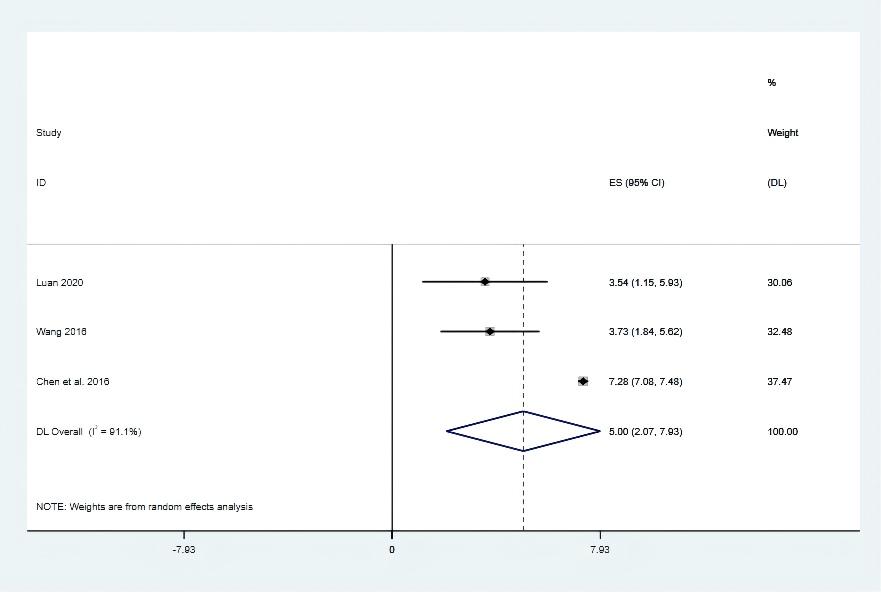


**Supplementary Datasheet 2.6.** Results of conventional meta-analysis，BBS, TCEP


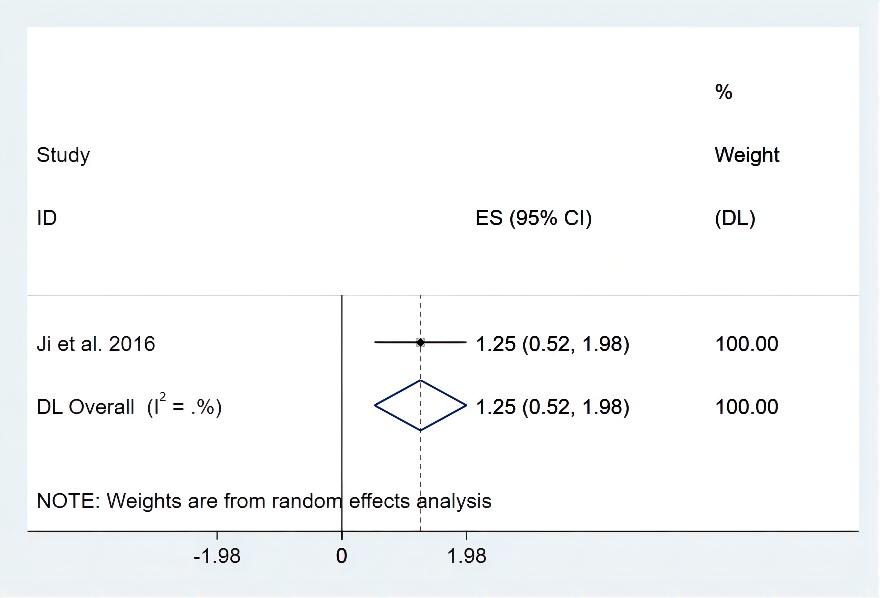


**Supplementary Datasheet 2.7.** Results of conventional meta-analysis，BBS, 8-form CS
